# Augmenting cost-effectiveness in clinical diagnosis using extended whole-exome sequencing: SNVs, SVs, and beyond

**DOI:** 10.1038/s10038-025-01403-4

**Published:** 2025-09-08

**Authors:** Fuyuki Miya, Daisuke Nakato, Hisato Suzuki, Mamiko Yamada, Daisuke Watanabe, Toshiki Takenouchi, Kenjiro Kosaki

**Affiliations:** 1 Center for Medical Genetics, Keio University School of Medicine, Tokyo, Japan; 2 Department of Clinical Medicine, Institute of Medicine, University of Tsukuba, Ibaraki, Japan; 3 Department of Pediatrics, Yamanashi University, Yamanashi, Japan; 4 Department of Child Neurology, Okayama University Graduate School of Medicine, Dentistry and Pharmaceutical Sciences, Okayama, Japan

**Keywords:** Genetics research, Disease genetics

## Abstract

In standard short-read whole-exome sequencing (WES), capture probes are typically designed to target the protein-coding regions (CDS), and regions outside the exons—except for adjacent intronic sequences—are rarely sequenced. Although the majority of known pathogenic variants reside within the CDS as nonsynonymous variants, some disease-causing variants are located in regions that are difficult to detect by WES alone, such as deep intronic variants and structural variants, often requiring whole-genome sequencing (WGS) for detection. Moreover, WES has limitations in reliably identifying pathogenic variants within mitochondrial DNA or repetitive regions. Here, we propose a strategy to improve the diagnostic yield in a cost-effective manner by expanding the target design of WES beyond the CDS. As an illustrative example, we experimentally validated an extended WES approach covering intronic and untranslated regions (UTRs) of 188 genes listed in the Japanese public health insurance-covered multiple gene testing, intronic and UTRs of 81 genes listed in ACMG Secondary Findings (SF) v3.2, and 70 repeat regions associated with diseases. Additionally, the entire mitochondrial genome was targeted. We demonstrate the coverage of these extended regions based on experimental data and present case examples in which previously diagnosed pathogenic variants located outside the CDS were successfully detected using this approach. This strategy enables a substantial increase in the chance of achieving a definitive diagnosis for patients using WES alone, without requiring WGS, at a cost comparable to conventional WES. Our method has the potential to significantly shorten the diagnostic odyssey and represents a valuable approach in clinical genomics.

## Introduction

More than 95% of known pathogenic variants are nonsynonymous variants that cause amino acid changes in protein-coding sequences (CDS) regions, including missense, nonsense, splicing site variants, and frameshift indels [1] (ClinVar, accessed May 1, 2025). Therefore, whole-exome sequencing (WES), which targets only the CDS regions of all genes, has been widely adopted worldwide as a cost-effective approach for identifying disease-causing variants. Although advances in next-generation sequencing (NGS) technologies have exponentially reduced the cost of genome sequencing [2], whole-genome sequencing (WGS) still costs more than twice as much as WES. As a result, many research institutions and clinical laboratories continue to utilize targeted resequencing or WES, focusing on specific genes, rather than WGS.

However, a subset of pathogenic variants resides outside the regions typically targeted by conventional WES, such as variants located within intronic regions, particularly those in deep intronic region, untranslated regions (UTRs), and structural variants (SVs) that are difficult to detect by WES and require WGS for accurate identification. For instance, deletions involving a single small exon with breakpoints located distantly within introns, or inversions including exons but with breakpoints in intronic or intergenic regions, are challenging to detect using standard WES.

In addition, WES often fails to detect pathogenic variants located in mitochondrial or repetitive regions. Recent advances have demonstrated that mitochondrial genotype information can be derived from off-target DNA sequences, including those outside the intended target regions of WES [3]. However, this approach remains underutilized. Furthermore, most commercially available WES capture kits are designed to exclude the mitochondrial genome DNA (mtDNA), resulting in inconsistent enrichment of mtDNA sequences [4]. This low and uneven coverage of off-target reads critically limits the reliable detection of mitochondrial-specific heteroplasmy, particularly for variants with low variant allele frequencies (VAFs).

Repetitive regions also pose a significant technical challenge for accurate sequencing by WES. To date, approximately 70 genes have been identified in which repeat expansions cause neuromuscular and other hereditary disorders [5–10]. However, these repeat regions are not necessarily located within CDS regions, and there is currently no standardized approach for detecting repeat expansions using WES. Thus, if pathogenic variants located outside CDS regions could be identified using WES without the need for WGS, this approach would represent a highly cost-effective strategy with significant utility in clinical diagnostics.

Here, we propose a novel strategy to improve the diagnostic yield of WES in a cost-effective manner by expanding the target regions beyond CDS. The selection of target genes depends on the clinical context of each patient. One effective strategy is to select genes relevant to each medical specialty, thereby covering disease-associated genes appropriate for patients in each clinical department. In this study, we designed extended target regions as an example of this strategy, which includes the following regions: (1) intronic and UTRs regions of genes covered by multi-gene testing for rare and intractable diseases reimbursed under the Japanese public health insurance system since June 2024 [11]; (2) repeat regions of genes known to cause disease through repeat expansions; (3) intronic and UTRs regions of genes important for reporting secondary findings; and (4) the mtDNA. We experimentally validated this approach, demonstrating the coverage of the expanded regions. Furthermore, we present successful cases of detecting pathogenic variants located outside CDS regions that had been identified in previous diagnostic cases.

## Methods

### Patients and participants

The experimental protocol was approved by the Ethics Committees of Keio University School of Medicine (approval No. 20110262 and 20211032). The methods were carried out in accordance with the approved guidelines. Written informed consent was obtained from the patients and their parents for the molecular studies. Genomic DNA was extracted from peripheral blood using standard protocols. As controls, DNA samples of HG001 (NA12878) and HG002 (NA24385), which are widely used as human reference genomes for benchmarking purposes, including in the Genome in a Bottle (GIAB) consortium [12], were obtained from the Coriell Institute.

### Custom capture probes

For the genomic regions of genes selected in this study, the design and synthesis of capture probes were performed by Twist Bioscience. For mitochondrial DNA, a commercially available kit, the Twist Mitochondrial Panel Kit (Twist Bioscience, South San Francisco, CA, USA), was used. The BED-format files specifying the target regions for all probes are provided in Supplementary Table 1.

### Genomic analysis

Targeted DNAs were captured using Twist Exome 2.0 plus Comprehensive Exome spike-in (Twist Bioscience) probes or further custom probes added to them, in accordance with the manufacturer’s instructions. The hybridization was performed using the “Fast protocol”, with an actual hybridization time of 90 minutes. The sequencing library was constructed using Twist Library Preparation EF Kit 2.0 according to the manufacturer’s instructions. The library DNAs were sequenced using Illumina NextSeq 500 (Illumina, San Diego, CA, USA). All sequencing was performed using 150 bp paired-end reads. Data analysis was performed as previously described [13]. In brief, single-nucleotide variants (SNVs) and insertions/deletions (indels) were called using GATK v4.5.0.0 following the GATK Best Practices workflow [14]. Structural variants (SVs) were detected using both Illumina DRAGEN (v4.3) and CNVkit [15]. Repeat expansions in the WES data were detected using ExpansionHunter [16] and visualized using STRipy (REViewer) [17]. When normalization of sequencing depth across samples was required for validation, seqkit [18] was used. For HG001 and HG002 samples, the high-confidence variant set v4.2.1 [19] from the GIAB consortium (https://www.nist.gov/programs-projects/genome-bottle) was used as the benchmark truth dataset.

For comparison with WGS data, DNA from HG001 and HG002 was used as input to prepare libraries using the Illumina DNA PCR-Free Prep Kit, following the manufacturer’s protocol. The libraries were sequenced on an Illumina NovaSeq 6000 platform with 151 bp paired-end reads. Variant calling was performed using Illumina DRAGEN v4.0.3.

### Statistical evaluation of variant detection accuracy

Statistical evaluation of variant detection accuracy was performed as previously described [13]. Briefly, recall (also referred to as sensitivity), precision, and *F1* score were calculated using the following formulas:

$${Recall}\,\left({sensitivity}\right)=\frac{{True\; Positive}\,({TP})}{{True\; Positive}\,\left({TP}\right)+{False\; Negative}\,({FN})}\,,\,$$

$${Precision}=\frac{{True\; Positive}\,({TP})}{{True\; Positive}\,\left({TP}\right)+{False\; Positive}\,({FP})}\,,\,$$

$$F1\,{score}=2\times \frac{{Precision}\times {Recall}}{{Precision}+{Recall}}\,.\,$$

## Results

### Design of extended capture probes

In this study, we designed an expanded set of custom capture probes to target genomic regions that are not adequately covered by conventional WES, which primarily focuses on CDS. These expanded regions included: (1) intronic and UTRs of genes listed in the multigene panel testing for rare and intractable diseases reimbursed under the Japanese public health insurance system as of June 2024 (hereafter referred to as “J-insurance”) [11]; (2) intronic and UTRs of genes recommended for reporting as secondary findings; (3) repeat regions known to be associated with disease; and (4) the full-length mitochondrial genome. For the J-insurance, we extracted 188 target genes from publicly available gene lists [11, 20] and designed probes for their intronic and UTRs. For secondary findings genes, we selected 81 genes from the ACMG SF v3.2 list [21] and designed probes to capture their intronic and UTRs. Disease-associated repeat regions were identified by integrating information from multiple sources [5–10], resulting in 70 genes for which probes were designed to capture known pathogenic repeat loci. For mitochondrial DNA, we employed a commercially available probe set (see METHODS). The complete list of genomic regions targeted by all probes is provided as a BED format file in Supplementary Table S1.

### Verification of the probe mixing ratio

We evaluated the optimal probe mixing ratios for the additional custom targets described above, which were added to the primary whole-exome capture probes targeting CDS. The intronic and UTRs of the 188 genes of J-insurance accounted for 8.6 Mb, corresponding to 22.9% of the total size of the exome regions (Table 1). Given that the primary goal of capturing intronic and UTRs was to enable the detection of large SVs, rather than achieving high coverage for accurate single nucleotide variants (SNVs) calling, we designed the study under the assumption that lower sequencing depth would be sufficient. To determine an appropriate probe concentration, we tested a series of probe mixing ratios relative to the main exome probe set: equal volume (×1), half (×0.5), one-quarter (×0.25), and one-tenth (×0.1). The proportion of bases covered at ≥10 depth, a threshold generally sufficient for variant detection, was comparable at ×1, ×0.5, and ×0.25; however, at ×0.1, the coverage at ≥10 depth decreased markedly (Supplementary Figs. S1, S2 and Supplementary Table S2). Based on these results, we selected ×0.25 as the mixing ratio for the intronic and UTRs probes of the J-insurance genes. Using the same design principle, we similarly evaluated the probe concentration for intronic and UTRs of the ACMG SF genes. The results supported the use of ×0.25 as the optimal mixing ratio for these probes as well (Supplementary Figs. S3 and S4, and Supplementary Table S3).

**Table 1:** Composition of the target regions in the extended WES design

Targeted region	Probes concentration ratio	Number of genes^1^	Targeted size (kb)
Exome (Twist Exome 2.0 plus Comprehensive Exome Spike-In)	$\times$1.0	20 584	37 453.1
Intronic and UTRs of J-insurance genes	$\times$0.25	188	8 594.7
Intronic and UTRs of ACMG SF v3.2 genes	$\times$0.25	81	3 971.5
Known pathogenic repeat expansion regions	$\times$1.0	70	5.9
Mitochondrial DNA (whole)	$\times$0.025	37	16.6

^1^Includes genes other than protein-coding genes

**Fig. 1:**
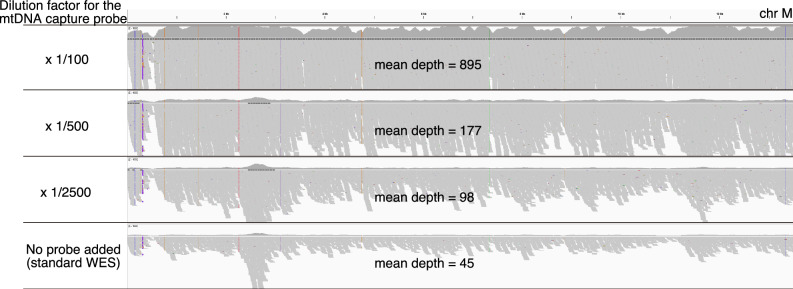
Coverage depth across the mitochondrial genome at various capture probe concentrations. Each gray bar represents a sequencing read. The numbers on the left indicate the dilution factor of the mitochondrial capture probes relative to the standard WES probes. The sequencing yields for each WES sample, from top to bottom, were 11.54 Gb, 10.18 Gb, 12.30 Gb, and 9.50 Gb, respectively. The figure was visualized using the Integrative Genomics Viewer (IGV)

Next, we evaluated the probe concentration for regions other than intronic and UTRs. For the repeat regions, the target size was relatively small; therefore, the probe concentration was set to be equivalent to that of the primary WES probes (Table 1). mtDNA is present in significantly higher copy numbers within cells compared to nuclear genomic DNA. For example, cardiac myocytes contain approximately 7000 times more mtDNA per cell, while peripheral blood cells contain approximately 100 times more mtDNA [22]. The proportion of captured mitochondrial sequences reflects the relative abundance of mtDNA within the total DNA extract. When mitochondrial probes are used at concentrations comparable to WES probes, the sequencing depth of mtDNA becomes disproportionately high. This imbalance reduces coverage of other target regions and decreases sequencing efficiency. To address the issue of probe concentration, this study investigated the optimal probe concentration for mtDNA sequencing using DNA extracted from the HG001 lymphoblastoid cell line (LCL). In the absence of mitochondrial-specific probes, the average depth of mtDNA was 45. As the amount of mitochondrial probes increased, the average mtDNA depth correspondingly increased (Fig. 1). DNA derived from LCLs tended to yield higher mtDNA depth compared to peripheral blood-derived DNA when using the same concentration of mitochondrial probes (data not shown). This is consistent with a previous report indicating that the mtDNA copy number in LCLs is approximately ten times higher than that in blood-derived mitochondria [22]. In addition, consistent with the previous reports, we have also observed that coverage tends to vary considerably depending on the DNA extraction method, the age of the sample donor, and inter-individual differences. Based on these observations, we determined that, for peripheral blood-derived DNA, adding mitochondrial probes at ×0.025 the concentration of the primary WES probes achieves a moderate and appropriate coverage depth—ranging from several hundred to just under 2000—neither too low nor excessively high (Table 1). Using the above probe mixing ratios, we prepared an “extended WES” probe set and conducted the following additional evaluations.

### Assessment of the extended WES coverage

**Fig. 2:**
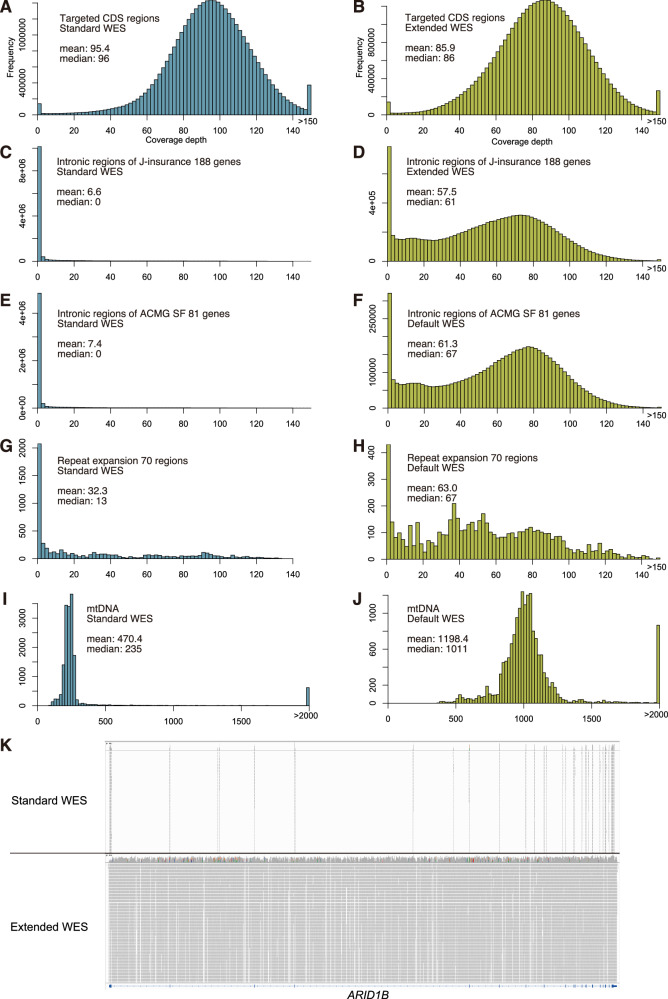
Distribution of coverage depth across different genomic regions in standard and extended WES. All WES datasets were normalized to 9 Gb of sequencing data prior to comparative analysis. **A**, **C**, **E**, **G**, **I** Coverage depth distribution for each genomic region in standard WES. **B**, **D**, **F**, **H**, **J** Coverage depth distribution for each genomic region in extended WES. **K** Comparison of sequencing reads between standard and extended WES for the *ARID1B* gene, visualized using the IGV

Using the standard genome DNA samples HG001 and HG002, we performed a comparative evaluation of coverage between standard WES and the extended WES. For comparison, the number of reads was normalized to 60 million reads (9 Gb with 150 bp paired-end) per sample prior to analysis. We assessed coverage metrics for five categories of target regions: (1) CDS regions targeted by standard WES probes, (2) intronic and UTRs of 188 J-insurance genes, (3) intronic and UTRs of 81 genes listed in the ACMG SF v3.2 list, (4) repeat regions from 70 disease-associated genes, and (5) the mitochondrial genome. The results for HG001 are shown in Fig. 2 and Supplementary Table S4. When normalized to the same sequencing reads (9 Gb), the average coverage depth over CDS regions was 9.5 (10.0%) lower in the extended WES compared to the standard WES. However, no notable differences were observed between the two methods in terms of the proportion of bases with depth >0, ≥10, or ≥20 (Fig. 2A, B, and Supplementary Table S4). For the intronic and UTRs, which were largely uncovered in standard WES, we defined coverage as regions with depth ≥10 and found that adding custom probes enabled coverage of over 85% of these regions (Fig. 2C–F and Supplementary Table S4). For the 70 repeat regions, although some were already covered in the CDS-targeted WES design, the majority showed limited coverage (~50%) with WES alone. However, with the addition of custom probes for repeat regions, coverage at depth ≥10 increased to over 85% (Fig. 2G, H, and Supplementary Table S4). For mitochondrial DNA, a marked increase in depth was observed, and coverage reached 100% even at the stringent threshold of depth ≥200 (Fig. 2I, J, and Supplementary Table S4). The same analysis was conducted for HG002, yielding results highly consistent with those of HG001, thereby confirming reproducibility (Supplementary Fig. S5 and Supplementary Table S4). Visual inspection using the Integrative Genomics Viewer (IGV) further confirmed that sequencing reads were broadly distributed across the intronic and UTRs of the target genes, resembling the coverage seen in WGS (Fig. 2K).

### Assessment of small variants calling with the extended WES

To evaluate the extent to which variant detection improves with the extended WES design, we compared variant calling in HG001 and HG002 against the high-confidence variants set provided by the GIAB consortium [19]. Variant calling of standard and extended WES was also performed using the same datasets, normalized to 9 Gb of sequencing data, as described above. As a reference for comparison, WGS was performed for HG001 and HG002, yielding 119.7 Gb and 126.5 Gb of sequencing data, respectively. Variant calling of WGS was then conducted, followed by comparison with the GIAB high-confidence variant set in the same manner. Four target regions were assessed: the CDS and the intronic+UTRs of the 188 J-insurance genes, and the CDS and intronic+UTRs of the 81 genes in the ACMG SF v3.2 list. Repeat regions were excluded from evaluation due to their small target size and the lack of corresponding high-confidence variants in GIAB. Similarly, mitochondrial DNA was excluded because it is not covered in the GIAB high-confidence variant set. For both HG001 and HG002 combined, the evaluation covered 1,090,149 bp and 614 variants in the CDS regions of the 188 insurance-covered genes, 15,188,849 bp and 18,213 variants in their intronic+UTRs, 672,783 bp and 323 variants in the CDS regions of the 81 ACMG SF genes, and 10,844,870 bp and 15 305 variants in their intronic+UTRs (Table 2).

**Table 2:** Analytical validation for standard WES and extended WES

Sequencing methods and samples	Benchmark region size (bp)	# High confidence SNVs and indels	Recall	Precision	*F1* score
J-insurance CDS for standard WES of HG001	535,105	297	0.980	0.997	0.988
J-insurance CDS for standard WES of HG002	555,044	317	0.981	0.997	0.989
J-insurance CDS for extended WES of HG001	535,105	297	0.980	0.993	0.986
J-insurance CDS for extended WES of HG002	555,044	317	0.984	0.997	0.990
J-insurance CDS for extended WGS of HG001	535,105	297	0.966	0.997	0.981
J-insurance CDS for extended WGS of HG002	555,044	317	0.994	1.000	0.997
J-insurance intronic+UTRs for standard WES of HG001	7,562,731	8900	0.242	0.864	0.378
J-insurance intronic+UTRs for standard WES of HG002	7,626,118	9313	0.224	0.857	0.355
J-insurance intronic+UTRs for extended WES of HG001	7,562,731	8900	0.909	0.925	0.917
J-insurance intronic+UTRs for extended WES of HG002	7,626,118	9313	0.905	0.921	0.913
J-insurance intronic+UTRs for extended WGS of HG001	7,562,731	8 900	0.983	0.991	0.987
J-insurance intronic+UTRs for extended WGS of HG002	7,626,118	9 313	0.990	0.991	0.990
ACMG SF genes CDS for standard WES of HG001	326,938	161	0.994	1.000	0.997
ACMG SF genes CDS for standard WES of HG002	345,845	162	1.000	0.988	0.994
ACMG SF genes CDS for extended WES of HG001	326,938	161	0.994	1.000	0.997
ACMG SF genes CDS for extended WES of HG002	345,845	162	1.000	1.000	1.000
ACMG SF genes CDS for extended WGS of HG001	326,938	161	0.975	0.994	0.984
ACMG SF genes CDS for extended WGS of HG002	345,845	162	1.000	1.000	1.000
ACMG SF genes intronic+UTRs for standard WES of HG001	5,397,183	7399	0.185	0.841	0.303
ACMG SF genes intronic+UTRs for standard WES of HG002	5,447,687	7906	0.158	0.851	0.267
ACMG SF genes intronic+UTRs for extended WES of HG001	5,397,183	7399	0.910	0.936	0.923
ACMG SF genes intronic+UTRs for extended WES of HG002	5,447,687	7906	0.912	0.921	0.916
ACMG SF genes intronic+UTRs for extended WGS of HG001	5,397,183	7399	0.988	0.991	0.989
ACMG SF genes intronic+UTRs for extended WGS of HG002	5,447,687	7906	0.991	0.991	0.991

The recall, precision, and *F1* score for each region are summarized in Table 2. Overall, there was little difference in performance between the standard WES and the extended WES in CDS regions. In contrast, for the intronic+UTRs, the recall markedly improved from approximately 0.2 with standard WES to over 0.9 with the extended WES, indicating a substantial increase in true positive detection and overall sensitivity. Precision in the intronic+UTRs also improved by approximately 0.1, suggesting a reduction in false positives. The *F1* score, which reflects the balance between recall and precision, showed a notable difference between the standard and extended WES in these non-CDS regions—rising from approximately 0.3 with standard WES to over 0.9 with the extended WES (Table 2).

### Detection of pathogenic variants with the extended WES

**Fig. 3:**
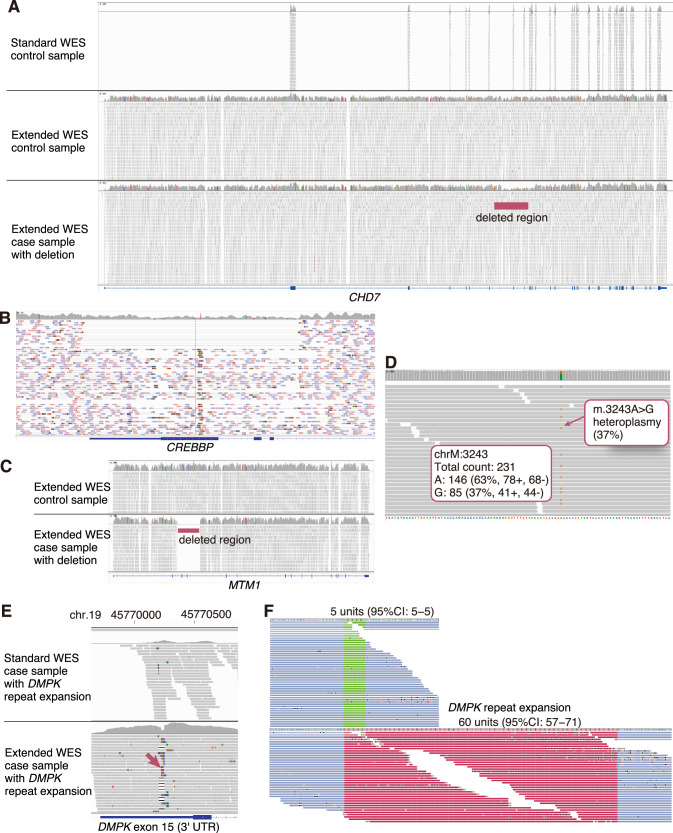
Pathogenic variants detected by extended WES. **A** A case with heterozygous deletion in the *CHD7* gene region. From top to bottom: standard WES in a control, extended WES in a control, and extended WES in the deletion case. **B** A case with a heterozygous deletion in the *CREBBP* gene. **C** A case with a homozygous deletion in the *MTM1* gene. **D** A case with heteroplasmy of mitochondrial m.3243 A > G variant. **E** A case with a heterozygous repeat expansion in the *DMPK* gene. The top panel shows standard WES data, and the bottom panel shows extended WES data. In the region indicated by the arrow, a portion of the sequence inconsistent with the reference genome, corresponding to the repeat expansion, is observed as clipped reads. **F** Visualization of the *DMPK* repeat expansion region using the STRipy tool

Finally, we evaluated the utility of the extended WES using samples known to harbor pathogenic SVs and SNVs. In a sample with a heterozygous 10.5 kb deletion spanning from intron 7 to intron 12 of the *CHD7* gene, which we previously reported [23], a marked reduction in coverage depth across the deleted region was clearly visible upon visual inspection using IGV, and the deletion was successfully detected by SV-calling tools (Fig. 3A). Similarly, a heterozygous 14.5 kb deletion from intron 21 to intron 28 (chr16 (NC_000016.10):g.3732368_3746832del) of *CREBBP* gene was accurately identified (Fig. 3B). A homozygous 9.3 kb deletion spanning intron 2 to intron 4 (chrX (NC_000023.11):g.150594723_150604022del) of *MTM1* gene was also readily detected (Fig. 3C). We also successfully identified a mitochondrial m.3243 A > G variant with approximately 37% heteroplasmy in a sample (Fig. 3D). The heteroplasmy level in this case had been independently validated as 38% using digital PCR (data not shown), demonstrating a high concordance between methods. In addition, a clear increase in coverage depth was observed in the 3′ UTR of the *DMPK* gene—one of the targeted repeat expansion loci (Fig. 3E). Notably, in the sample with an undetected repeat expansion using standard WES, the expanded repeat was successfully identified using the extended WES (Fig. 3F). The repeat expansion in *DMPK* was confirmed by Oxford Nanopore long-read sequencing to consist of approximately 1500 to 8500 bp of expanded CAG repeats (Supplementary Fig. S6).

## Discussion

In this study, we proposed a cost-effective strategy to improve diagnostic yield by expanding the target regions of WES. While the cost of WGS has declined in recent years, it still remains approximately twice as expensive as WES. Therefore, extended WES offers not only a more economical alternative, but also increases the likelihood of achieving a definitive diagnosis using WES alone. This reduces the need for subsequent WGS after a negative WES result, thereby shortening the diagnostic odyssey. The strategy can be adapted to various clinical specialties—for instance, a gene panel for hearing loss, for retinitis pigmentosa, or for epilepsy—depending on the expertise of the ordering physician. What we particularly aimed to emphasize in this study is the strategy of including intronic regions to enable the detection of SVs. We selectively expanded the target regions to include the intronic and UTRs of clinically important genes listed in the J-insurance and ACMG SF as an example of this strategy, while retaining the standard WES design that targets only the CDS for other genes. If targeted resequencing were limited solely to the full length of these approximately 300 selected genes, pathogenic variants in other genes would be missed, potentially prolonging the diagnostic odyssey. On the other hand, expanding the target to cover intronic and UTRs of all genes would drastically increase the target size (~30% of whole-genome) and severely compromise cost-effectiveness, making such an approach impractical. Therefore, we selected the target regions used in this study as a balanced and efficient strategy to maximize diagnostic yield while maintaining cost-effectiveness. Furthermore, extended WES also offers advantages over WGS in terms of reduced time and cost for data storage and analysis.

The total size of the additional target regions introduced in this study was 12.6 Mb (Table 1), which is substantial. Notably, 98.8% of the added bases correspond to intronic and UTRs of J-insurance and ACMG SF genes. To minimize the increase in required sequencing data due to this target expansion, we adopted a probe mixing ratio of ×0.25 relative to the standard WES probe concentration for intronic and UTRs probes. As shown in our results, this reduced probe concentration was sufficient for detecting SVs within intronic regions. Consequently, even though the target region size increased by 1.34-fold, the total required sequencing data increased by only approximately 1.2-fold. This indicates that our design preserves the multiplexing capacity of sequencing runs, allowing for efficient processing of multiple samples per run. As shown in Fig. 2A and B, when normalized to the same sequencing reads, the coverage depth over CDS regions was 9.5 (10.0%) lower in the extended WES compared to the standard WES. However, as shown in Table 2, this difference in coverage did not affect the accuracy of variant detection within CDS regions at the same sequencing reads. Increasing the sequencing reads of the extended WES to 1.2 times that of the standard WES is expected to achieve a comparable average depth in CDS regions. Note that, while sequencing costs may vary depending on whether the experiment is performed in-house or outsourced, the type of sequencer used, and regional pricing of reagents, we estimate that the cost of extended WES is approximately 1.2 times that of standard WES. Therefore, compared with WGS, which typically costs more than twice as much as standard WES, extended WES can be considered a cost-effective approach.

A similar strategy of moderate coverage was also applied to mtDNA. Due to heteroplasmy, pathogenic mtDNA variants may exhibit high VAF in affected tissues but much lower VAF in blood. In some carriers, the VAF may not reach the threshold required for disease manifestation. Thus, we aimed to achieve a balanced mtDNA coverage depth in the range of several hundred reads—not too low to miss clinically relevant variants, but not unnecessarily high—allowing for reliable heteroplasmy detection while maintaining overall sequencing efficiency. Furthermore, the mitochondrial average depth may vary substantially depending on various factors, including the DNA extraction method. For example, although both Fig. 1 and Fig. 2I used HG001 DNA, the former employed DNA extracted in-house from cultured cells, whereas the latter used DNA purchased directly from the Coriell Institute, leading to a marked difference in mitochondrial coverage. Since standard WES without dedicated mitochondrial probes may result in insufficient depth and missed variant calls, including mtDNA-specific probes in an extended WES panel represents a practical strategy to enhance mitochondrial variant detection.

While it is self-evident that greater coverage depth improves detection sensitivity across the genome, the optimization of this parameter is critical when considering cost-effectiveness. In the early phase of the 1000 Genomes Project [24], the average coverage depth was 3.7×, and in the UK10K Project [25], it was approximately 7×. These large-scale population studies demonstrated that low-coverage sequencing can still achieve adequate sensitivity in population-level variant detection. Even for individual samples, it has been reported that a depth of approximately 15× is sufficient for accurate detection of SNVs [26]. In our validation, WGS with an average depth of approximately 30× demonstrated comparable sensitivity to WES ( ~ 90×) in coding regions, and superior sensitivity to extended WES in intronic and UTR regions (Table 2). This is likely due to the advantage of WGS in regions where probe design is challenging, such as redundant genomic sequences and high-GC content regions, as WGS does not rely on a capture step. In general, it is widely accepted that WGS requires a minimum average depth of 30×, while WES typically requires 50× to ≥100× coverage [27]. WES generally requires higher coverage depth than WGS due to uneven coverage caused by factors such as variation in GC content across the genome and challenges in probe design. Another contributing factor is that the ends of the capture targets—namely, exon boundaries—tend to show lower depth, necessitating higher average depth overall to maintain detection sensitivity in these critical boundary regions. In this study, our extended WES design mitigates this issue by including intronic regions in the probe design, which reduces the risk of decreased depth at exon-intron boundaries. As a result, even with a lower average sequencing depth, our design enables reliable detection of variants that affect splicing at exon-intron junctions.

Most importantly, our probe design strategy improved the detectability of SVs by covering intronic regions. In our previous study [13], we demonstrated that small SVs—particularly those involving only a single exon—are often missed by many SV detection tools, highlighting the need for improved sensitivity. With intronic sequences included, it becomes easier to detect abrupt changes in coverage across SV breakpoints. Furthermore, in cases such as intronic inversions where coverage over exons remains unchanged, the presence of reads spanning intronic breakpoints can still reveal the underlying SV event.

A distinctive feature of this study is the inclusion of repeat expansion regions known to cause repeat-associated disorders as part of the capture target. Due to the repetitive nature of these regions, designing capture probes directly within the repeat sequences is generally infeasible, making their inclusion technically challenging. To address this, we placed an increased number of probes flanking the repeat regions, which enabled the detection of several expansions with a reasonable degree of success. However, some repeat loci, such as the CGG repeat in the* FMR1* gene associated with Fragile X syndrome, exhibit extremely high GC content, making capture particularly difficult. In our study, such GC-rich repeat regions showed shallower coverage compared to other repeat-targeted regions, and this limitation should be acknowledged.

The tool used for repeat expansion detection in this study, ExpansionHunter [6], was originally developed for use with WGS. Nevertheless, we found that it can still detect certain repeat expansions using WES data. As demonstrated in the case of the *DMPK* gene, extremely long repeat expansions may not be accurately sized by short-read sequencing and may require confirmation by long-read sequencing technologies. However, deviations from the normal repeat range can often be identified even from short-read WES data, as shown in this study. Therefore, incorporating repeat expansion loci into the capture design for short-read WES represents a rational and feasible strategy to improve the detection of repeat-associated disorders.

In conclusion, we developed and present here a cost-effective strategy to enhance diagnostic yield and reduce turnaround time by extending WES to include intronic and UTRs of selected genes, repeat expansion loci, and the mitochondrial genome. This approach can be readily adapted to future changes in gene panels or disease coverage under public insurance schemes by updating the probe design accordingly. We hope that this strategy will be utilized in research and clinical settings and ultimately benefit patients through more efficient genetic diagnostics.

## Supplementary information

Supplementary Figure S1

Supplementary Figure S2

Supplementary Figure S3

Supplementary Figure S4

Supplementary Figure S5

Supplementary Figure S6

Supplementary Table S1

Supplementary Table S2

Supplementary Table S3

Supplementary Table S4

## Data Availability

The HG001 and HG002 sequencing data in this study are freely available in the DDBJ Sequencing Read Archive (SRA) under the accession numbers DRR635837, DRR635838, DRR635839, DRR635840, DRR717673, and DRR717674 (Project No. PRJDB20167). The other data of this study are available from the corresponding author upon reasonable request. The probe set designed in this study is available for purchase from Twist Bioscience under the name “Nanbyome” panel. We declare that we have received no financial benefit from Twist Bioscience regarding the sale of this panel.
