# Supplementary material for: Augmenting cost-effectiveness in clinical diagnosis using extended whole-exome sequencing: SNVs, SVs, and beyond
Source: J Hum Genet. 2025 Sep 8;71(1):13–21. doi: 10.1038/s10038-025-01403-4 (PMC12689423; doi:10.1038/s10038-025-01403-4)
Supplement: Supplementary file 1 — Supplementary Figure S1 [file 10038_2025_1403_MOESM1_ESM.pdf]

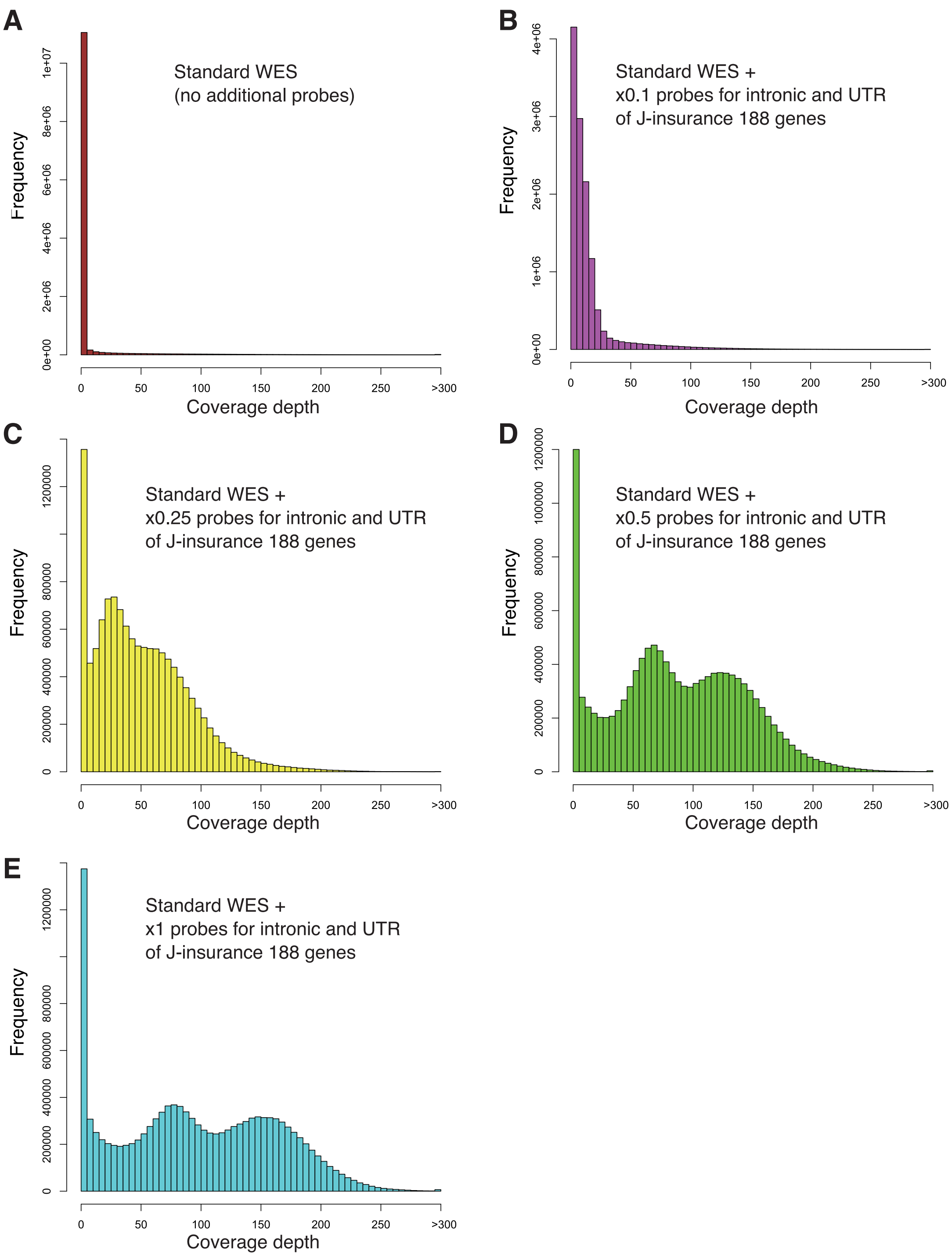


**Supplementary Fig. S1** Coverage depth distribution for probes targeting intronic and UTR regions of J-insurance-listed genes at different probe concentrations. The sequencing yields for each WES sample were as follows: 13.45 Gb (A), 8.90 Gb (B), 10.55 Gb (C), 11.28 Gb (D), and 9.90 Gb (E).
