# Supplementary material for: Augmenting cost-effectiveness in clinical diagnosis using extended whole-exome sequencing: SNVs, SVs, and beyond
Source: J Hum Genet. 2025 Sep 8;71(1):13–21. doi: 10.1038/s10038-025-01403-4 (PMC12689423; doi:10.1038/s10038-025-01403-4)
Supplement: Supplementary file 3 — Supplementary Figure S3 [file 10038_2025_1403_MOESM3_ESM.pdf]

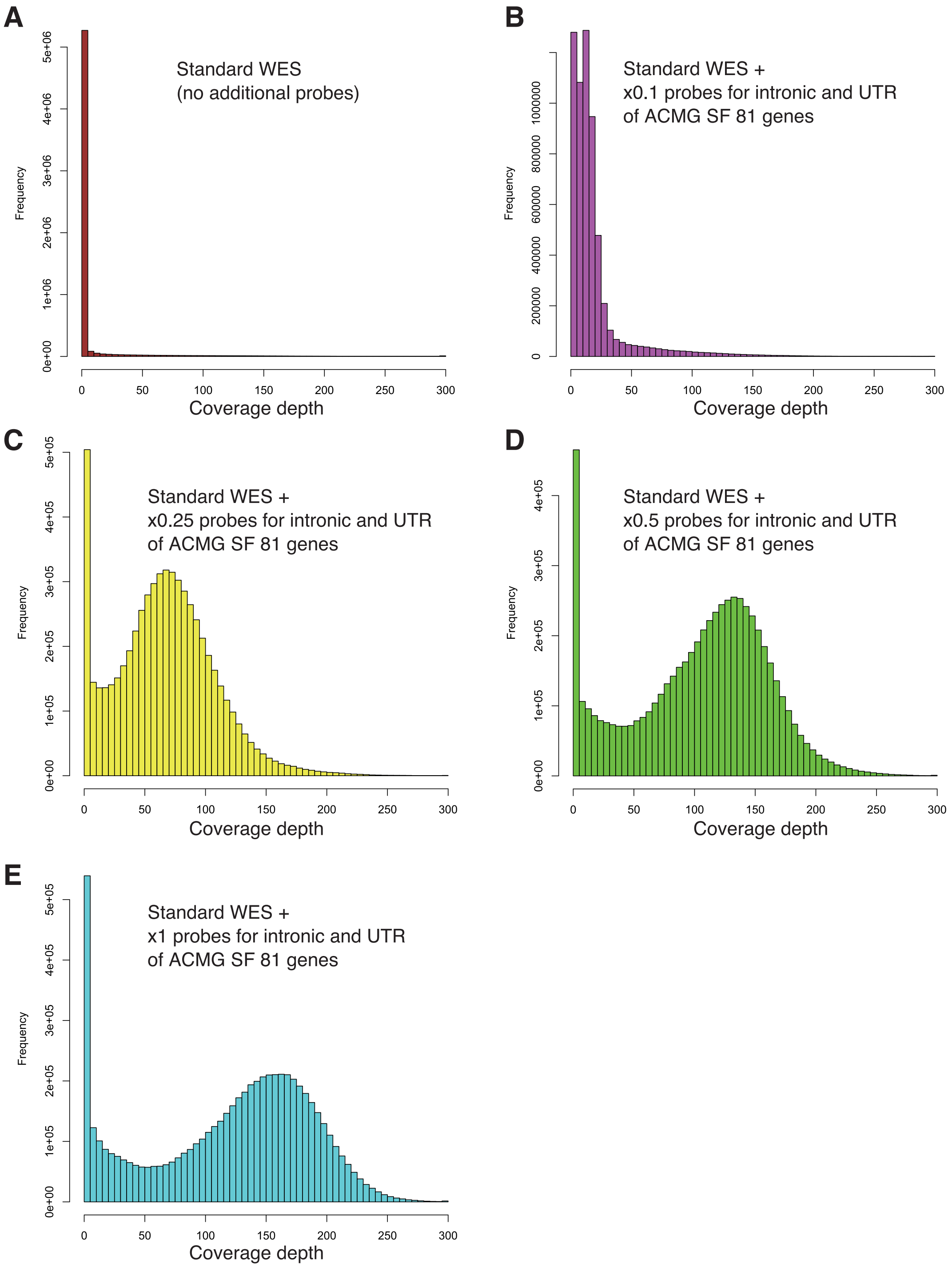

Supplementary Fig. S3 Coverage depth distribution for probes targeting the intronic and UTR regions of the 81 ACMG SF genes under different probe concentrations. The sequencing yields for each WES sample are the same as those described in **Supplementary Fig. S1**.
