# Supplementary material for: Augmenting cost-effectiveness in clinical diagnosis using extended whole-exome sequencing: SNVs, SVs, and beyond
Source: J Hum Genet. 2025 Sep 8;71(1):13–21. doi: 10.1038/s10038-025-01403-4 (PMC12689423; doi:10.1038/s10038-025-01403-4)
Supplement: Supplementary file 4 — Supplementary Figure S4 [file 10038_2025_1403_MOESM4_ESM.pdf]

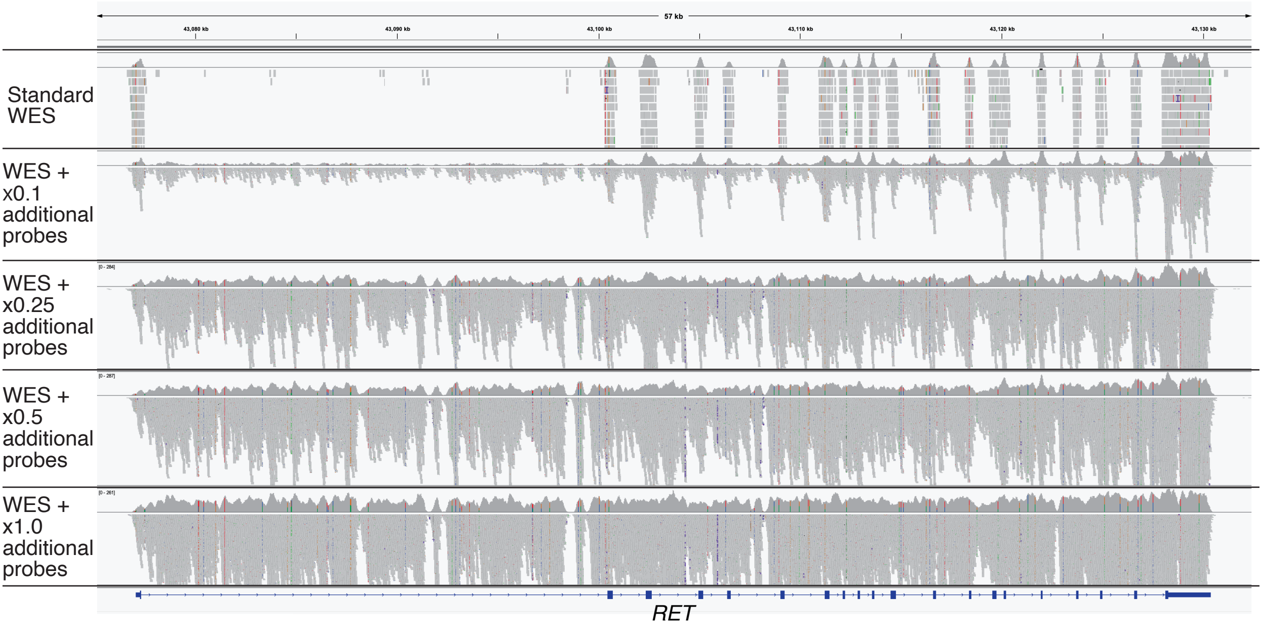

Supplementary Fig. S4 Distribution of coverage depth for the *RET* gene at various probe concentrations targeting the intronic and UTR regions of ACMG SF genes. *RET* is one of the genes included in the ACMG SF list. The figure shows the coverage distribution as visualized by the IGV, with each gray bar representing an individual sequencing read. The sequencing yields for each WES sample are the same as those described in **Supplementary Fig. S1**
