# Supplementary material for: Augmenting cost-effectiveness in clinical diagnosis using extended whole-exome sequencing: SNVs, SVs, and beyond
Source: J Hum Genet. 2025 Sep 8;71(1):13–21. doi: 10.1038/s10038-025-01403-4 (PMC12689423; doi:10.1038/s10038-025-01403-4)
Supplement: Supplementary file 5 — Supplementary Figure S5 [file 10038_2025_1403_MOESM5_ESM.pdf]

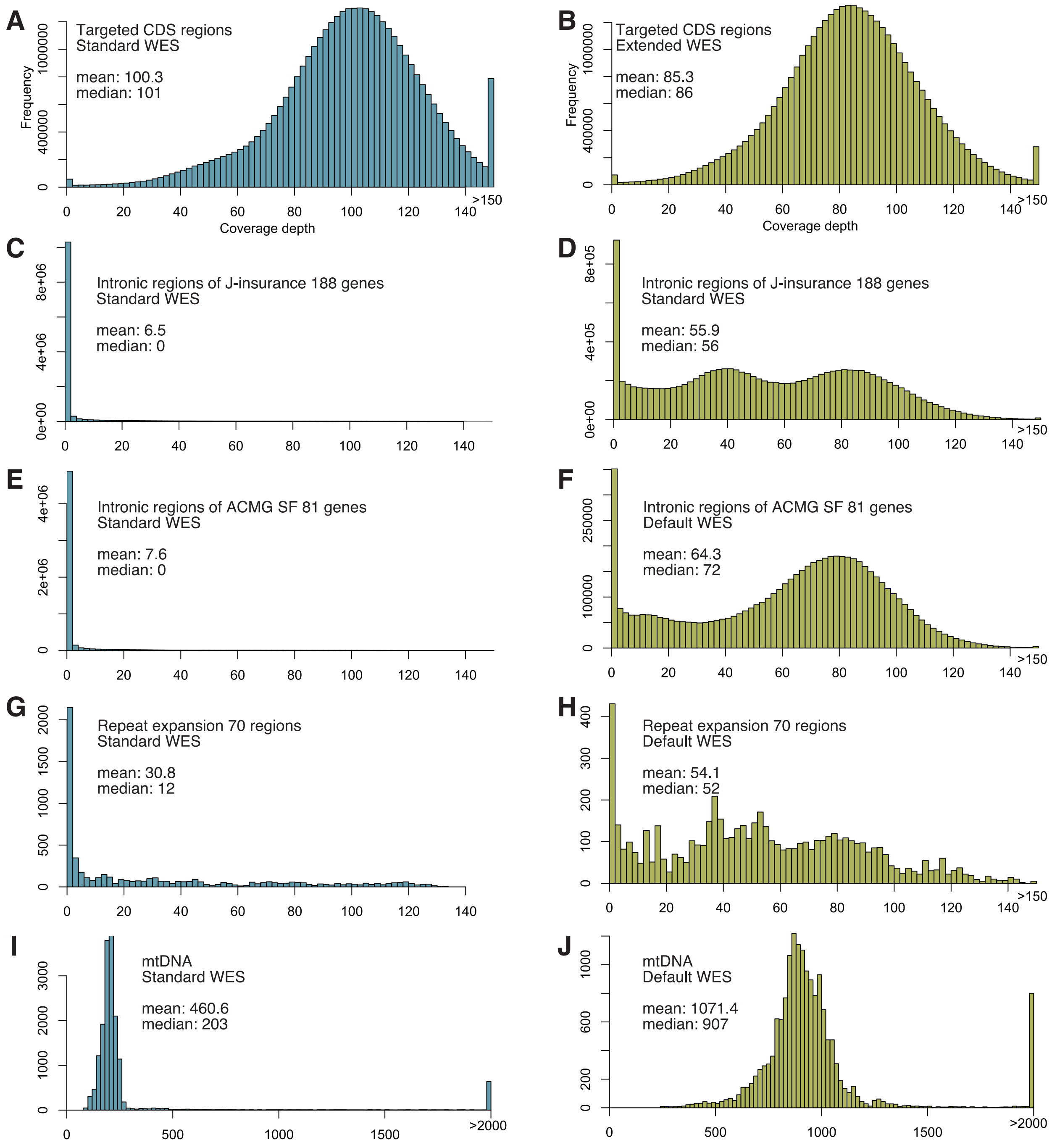

Supplementary Fig. S5 Distribution of coverage depth across different genomic regions in standard and extended WES for HG002 sample. All WES datasets were normalized to 9 Gb of sequencing data prior to comparative analysis. **A**, **C**, **E**, **G**, **I** Coverage depth distribution for each genomic region in standard WES. **B**, **D**, **F**, **H**, **J** Coverage depth distribution for each genomic region in extended WES
