# Supplementary material for: Augmenting cost-effectiveness in clinical diagnosis using extended whole-exome sequencing: SNVs, SVs, and beyond
Source: J Hum Genet. 2025 Sep 8;71(1):13–21. doi: 10.1038/s10038-025-01403-4 (PMC12689423; doi:10.1038/s10038-025-01403-4)
Supplement: Supplementary file 6 — Supplementary Figure S6 [file 10038_2025_1403_MOESM6_ESM.pdf]

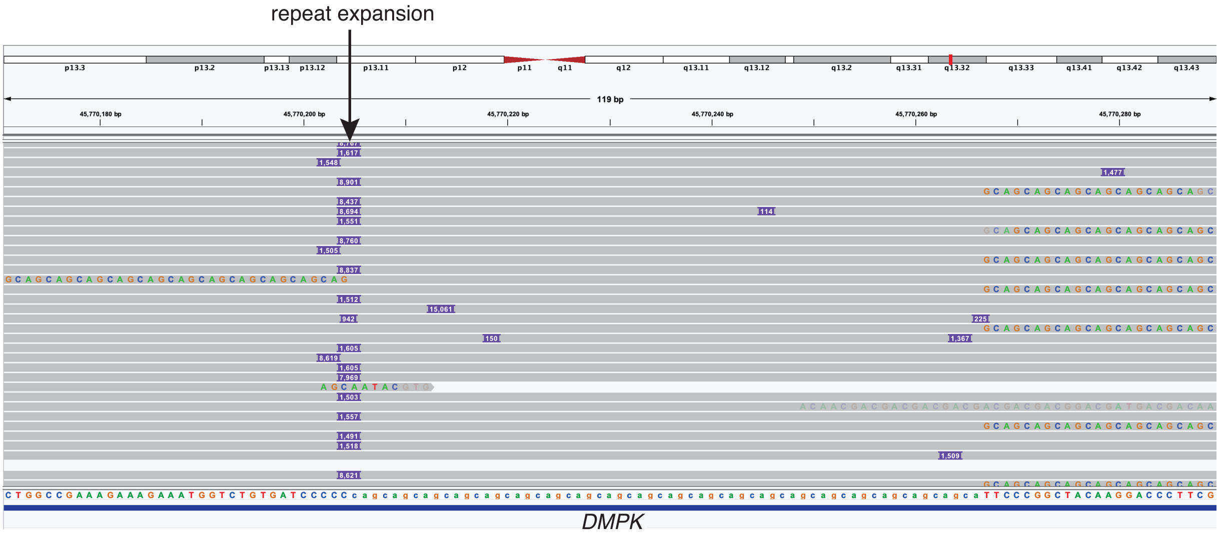

Supplementary Fig. S6 Validation of the 3′ UTR repeat expansion in *DMPK* by long-read sequencing. A sample exhibiting *DMPK* repeat expansion was sequenced using Oxford Nanopore long-read sequencing. Sequencing was performed on the PromethION 2 Solo device, and the sequencing library was prepared using the Library Ligation Kit V14 according to the manufacturer's protocol. Base calling was performed with Dorado v0.9.1, and the reads were mapped to the human reference genome (hg38) using Minimap2 (v.2.28). The figure visualizes the expanded region in the 3′-UTR of *DMPK* as viewed in IGV. The numbers enclosed in purple boxes indicate the number of expanded bases
